# Association Between Air Pollution and Acute Coronary Syndromes During Lockdown for COVID-19: Results From the Terni Hub Center

**DOI:** 10.3389/fpubh.2021.683683

**Published:** 2021-06-24

**Authors:** Alessio Arrivi, Marcello Dominici, Nicola Bier, Mauro Truglio, Gaetano Vaudo, Giacomo Pucci

**Affiliations:** ^1^Interventional Cardiology Unit, “Santa Maria” University Hospital, Terni, Italy; ^2^Department of Medicine and Surgery, University of Perugia, Perugia, Italy; ^3^Laboratory of Cutaneous Physiopathology, San Gallicano Dermatological Institute Istituto di Ricovero e Cura a Carattere Scientifico (IRCCS), Rome, Italy; ^4^Unit of Internal Medicine, “Santa Maria” University Hospital, Terni, Italy

**Keywords:** air pollution, acute coronary syndromes, invasive procedures, COVID-19, lockdown, particulate matter

## Abstract

**Background:** During the lockdown for COVID-19, a massive decrease in hospital admissions for acute coronary syndrome (ACS) and a drop in air pollution were both detected in Italy. Our aim was to investigate the possible association between these two events at the Province of Terni, one of the most polluted urban and industrial area in Central Italy.

**Methods:** We analyzed data of daily 24-h urban air concentrations of particulate matter (PM)10 and PM2.5 from fixed station monitoring network located in the main city centers of the Terni province, and accesses for ACS at the catheterization laboratory of the Cardiological Hub Center of the Terni University Hospital during lockdown. A comparison was made with data corresponding to the same lockdown time period of years 2019, 2018, and 2017.

**Results:** Invasive procedures for ACS decreased in 2020 (*n* = 49) as compared with previous years (*n* = 93 in 2019, *n* = 109 in 2018, and *n* = 89 in 2017, *p* < 0.001). Conversely, reductions in average PM10 (20.7 μg/m^3^) and PM2.5 (14.7 μg/m^3^) in 2020 were consistent with a long-term decreasing trend, being comparable to those recorded in 2019 and 2018 (all *p* > 0.05) and slightly lower than 2017 (*p* < 0.05). The Granger-causality test demonstrated the lack of association between time-varying changes in air pollution and the number of procedures for ACS.

**Conclusions:** Our results did not support the hypothesis that reduction in invasive procedures for ACS during lockdown was linked to an air cleaning effect. Reasons other than reduced air pollution should be sought to explain the observed decrease in ACS procedures.

## Introduction

The coronavirus disease 19 (COVID-19) drastically reduced access to healthcare in 2020. Specifically, during the lockdown period, hospital admissions for acute coronary syndrome (ACS) suddenly decreased in Italy. This decrease was found significant for ST-elevation myocardial infarction (STEMI) (27%) and Non-ST-elevation myocardial infarction (NSTEMI) (65%), both in the north and in the central/south of Italy (1). Similarly, an air cleaning effect was observed by NASA and European Space Agency pollution monitoring satellites (2).

Air pollution is a sophisticated mixture of gases and particulate components (PM10 and PM2.5 with aerodynamic diameter ≤10 and ≤2.5 μM, respectively), whose concentration may change depending on the source, emission rate, and climate conditions (3). In most medical research, air pollution had a significant relationship with the occurrence of ACS (4, 5). Data from systematic reviews and meta-analyses showed that short-term exposure to particulate matter (PM_2.5_ and PM_10_) is a significant risk factor for myocardial infarction (6). A 13% relative increase in non-fatal acute coronary events was observed over the long term following a 5 μg/m^3^ increase in estimated annual mean PM_2.5_ and a 10 μg/m^3^ increase in estimated annual mean PM_10_ (7). In a study carried out in the urban areas of Utah (USA), the exposure to fine particulate matter air pollution contributed to trigger acute coronary events in patients with CAD (8). Nevertheless, the accuracy, completeness, and representativeness of data largely vary across studies (5, 9, 10) and the matter remains controversial, given also the presence of discordant results showing no association (11–13). Therefore, examining this interplay during a unique and unprecedented social upheaval, such as lockdown for COVID-19 pandemic (14), may contribute to clarify the debate on the topic.

In this research report, we aimed at analyzing the relationship between the number of invasive procedures for ACS performed at the Cardiological Hub Center of Terni, one of the most polluted urban and industrial areas in Central Italy (15), and atmospheric air pollution trends measured during the lockdown period in comparison with previous years. The main hypothesis was to find evidence of an association between variations in air quality and the number of invasive procedures for ACS through the analysis of time series data that included the lockdown period.

## Materials and Methods

We retrospectively collected data related to admissions to the emergency service of the Cardiological Hub Center of the Terni University Hospital, which is equipped with catheterization laboratory providing the 24-h 7-days-a-week primary PCI (24/7 pPCI) service. This latter fully covers the cardiological emergencies of Terni and its province, a basin with about 226,000 inhabitants. We selected the number of patients who received a diagnosis of any ACS including unstable angina (UA, ICD-9 code 411.1), NSTEMI (ICD-9 code 410.7) and STEMI (ICD-9 code 410.9), and related invasive procedures, during the time period corresponding to the national lockdown, going from March 9, 2020, to May 3, 2020. To make comparison between years and to avoid running into a seasonality bias, we collected the same data during the same time period for the years 2019, 2018, and 2017. People residing outside the province of Terni and accessing the Cardiology Hub service for ACS during the periods under examination were excluded from the overall analysis. For this particular research project, individual data were not collected; data were protected for privacy purposes by rendering it anonymous prior to analysis.

**Figure 1:**
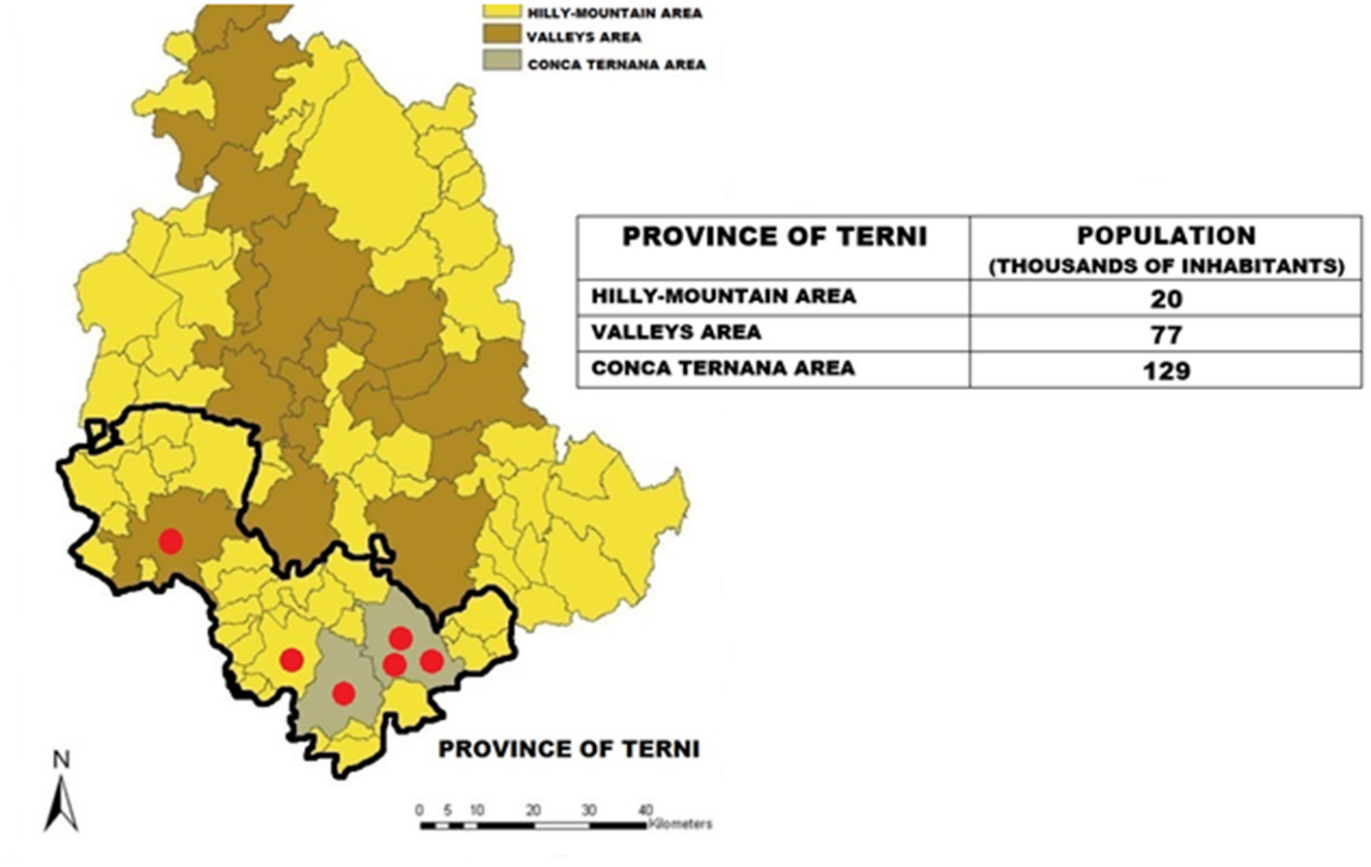
Geographical map of the province of Terni showing the distribution of the network of fixed public monitoring stations, consisting of six units located within the main city centers of the Terni province with more than 10,000 inhabitants (Terni = 3, Narni = 1, Amelia = 1, Orvieto = 1, total population 161,771 people, representing the 72% of the province of Terni).

We collected, on a daily basis and for the same period of time as described above, data about the average 24-h air concentration of air pollutants. Two particulate matter (PM) pollutants, PM_10_ and PM_2.5_, were selected for this analysis because they were shown to have a significant relationship with the occurrence of ACS in previous research (4, 5). These data were extracted from the data made public on the service website (www.arpa.umbria.it), and corresponded to the average value recorded from the entire network of fixed public monitoring stations, consisting of six units located within the main city centers of the province of Terni with more than 10,000 inhabitants (Terni = 3, Narni = 1, Amelia = 1, Orvieto = 1, total population 161,771 people, representing the 72% of inhabitants of the Province of Terni, Figure 1). The two PM measures (PM_10_ and PM_2.5_) were standardized and averaged out.

## Statistical Analysis

We first tested the normality of the distribution of pollutants (PM_10_ and PM_2.5_) through appropriate statistics (Kolmogorov–Smirnov test) and performed an analysis of variance for repeated measures (Repeated Measures ANOVA) to evaluate an overall difference in the distribution of average values across years. This test was combined with the appropriate *post-hoc* paired *t*-tests for further pairwise comparisons. The distribution of the daily number of procedures for ACS was analyzed through a Generalized Linear Model, which was checked for over- and underdispersion. The Iteratively Reweighted Least Squares (IRLS) algorithm was used in order to find the maximum likelihood estimate for the model (16), while the pairwise Wald test was used for *post-hoc* comparisons.

We performed an analysis of the significance between time series of both pollutants and number of procedures, based on three main steps: (1) a time/count analysis, to evaluate possible interactions between yearly time series of pollution and the number of procedures, by testing for at-a-glance visual similarities in slopes; (2) a dynamic time warping (17) (DTW), which measures a smart distance between two curves, taking any possible lags into account, from which it is possible to build dendrograms showing possible relations between all the elements under analysis; (3) the Granger causality test (18), which was run to evaluate possible causation relationships between the two object variables, properly treated as times series data whose stationarity was verified by the Augmented Dickey-Fuller method (19). According to its original formulation, a time series X(t) Granger-causes Y(t), if the past values of X(t) help in predicting the future values of Y(t). Similarly to the DTW, the Granger causality test takes into account possible lags between the two series under analysis. The optimal number of lags under study is usually a compromise between bias and power: using too few lags, there is a risk of a biased test because of residual auto-correlation; using too many could lead to potentially spurious rejections of the null hypothesis (i.e., random correlations). For this study, an interval of lags between 1 and 10 was tested. We carried out two sensitivity analyses: one after taking PM_10_ and PM_2.5_ separately, the second after restriction of the time span to 2019 and 2020, in order to find if a causation started during this time period.

## Results

**Figure 2:**
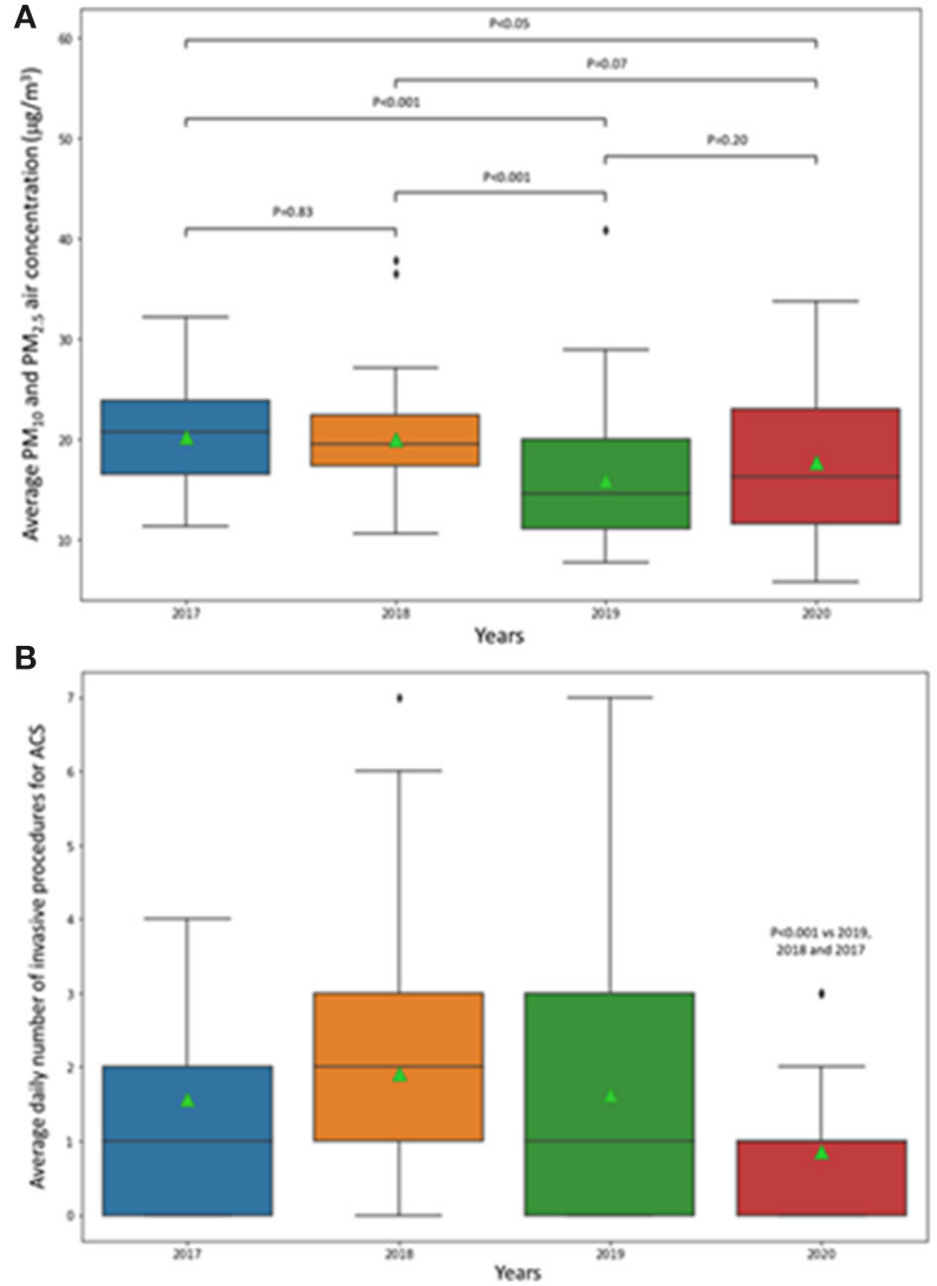
Box and whiskers plot representing **(A)** the distribution of particulate matter (PM)_10_ and PM_2.5_ air concentration and **(B)** the distribution of the number of invasive procedures for acute coronary syndromes (ACS) recorded between March 9 and May 3 of 2020, 2019, 2018, and 2017. The ends of the box are the upper and lower quartiles; the horizontal line inside the box represents the median, and the green triangle denotes the mean. The end of the whiskers represents the upper and lower extremes; data beyond those values are outliers.

The total number of invasive procedures for ACS, PM_10_ and PM_2.5_ levels and their average value, divided per years, are all reported in Table 1. Average PM_2.5_ and PM_10_ air concentrations recorded in 2020 were consistent with a long-term decreasing trend, being comparable with levels recorded during 2019 (15.9 mg/m^3^) and 2018 (20.1 mg/m^3^, *p* vs. 2020 >0.05). A slight, although significant, reduction was observed when levels recorded in 2020 were compared with 2017 (20.2 mg/m^3^, *p* vs. 2020 <0.05). PM_10_ and PM_2.5_ levels recorded during 2019 were also significantly lower than during 2018 and 2017 (both *p* < 0.001, Figure 2A).

**Table 1:** Average levels of particulate matter (PM)_2.5_ and PM_10_ (μg/m^3^), their average, and the number of invasive procedures for acute coronary syndromes (ACS) recorded during the lockdown period in 2020 and during the same time period in 2019, 2018, and 2017.

	**09/03/20–03/05/20**	**09/03/19–03/05/19**	**09/03/18–03/05/18**	**09/03/17–03/05/17**	**All**
PM_2.5_	14.7	12.6	15.0	16.3	14.6
PM_10_	20.7	19.3	25.1	24.2	22.3
Average	17.7	15.9	20.1	20.2	18.5
Invasive Procedures for ACS	49	93	109	89	340

Regarding the number of urgent invasive procedures for ACS, the GLM using further affirmation with IRLS algorithm revealed no signs of overdispersion (Pearson χ^2^/DF residuals = 1.32); accordingly, a significant reduction in procedures in 2020 as compared with the other years at the pairwise Wald test was found (*p* < 0.001, Figure 2B).

**Figure 3:**
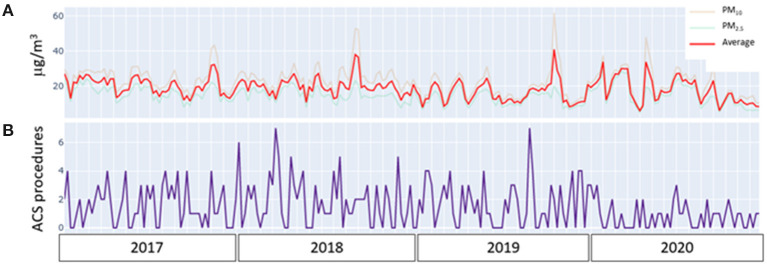
**(A)** Time/counts analysis plot of time series of air concentration of particulate matter (PM)_2.5_ (blue), PM_10_ (pink) air pollutants, and their average (red), in the examined periods from 2017 to 2020. **(B)** Time/counts analysis plot of time series of the number of invasive procedures for acute coronary syndromes (ACS procedures) in the examined periods from 2017 to 2020. The visual inspection of the graph excludes any at-a-glance similarity in slopes of the time series by years.

The analysis of the time series divided by years did not reveal any possible at-a-glance similarity in slopes of the time series for both procedures and air pollution during the whole time period explored (Figure 3).

In order to perform the Granger causality test, the stationarity of the time series was successfully tested with the Augmented Dickey–Fuller method (*p* = 0.005 for pollution, *p* < 0.000001 for invasive procedures count). The Granger test did not reveal any significant correlation between the time series describing the changes in air pollution and the number of procedures performed. This result demonstrated the absence of any relationship between changes in air pollution and the number of urgent procedures performed for ACS. In a sensitivity analysis, no differences were found when PM_10_ and PM_2.5_ air concentrations were analyzed separately, neither when the analysis was conducted considering the different ACS phenotypes (unstable angina, NSTEMI, and STEMI). Moreover, we did not find any significant difference in the overall results when the time series analysis was restricted only to years 2019 and 2020.

## Discussion

The main aim of our study was to assess possible associations between changes in particulate air components (PM_10_ and PM_2.5_) and the number of urgent invasive procedures for ACS managed at the Hub Center of the Terni University Hospital, covering the entire province of Terni (about 226,000 inhabitants), by analyzing the changes that took place during the time of the lockdown period imposed by the Italian government to contrast the consequences of the COVID-19 pandemic, compared to the same periods of time during the previous years. We decided to compare the same calendar period (March 9 to May 3) across the different years to avoid running into a seasonality bias: although the exact trigger of ACS may not always be readily apparent, seasonal variations in their incidence are well-known (20), with highest ACS events from March to May (21).

Our data showed only slight changes in air pollution levels of PM_10_ and PM_2.5_ recorded in 2020 across the Terni province. This trend was more consistent with a long-term improvement in air quality instead of a sudden decrease caused by restrictions imposed by the Italian government. Conversely, the reduction in urgent invasive procedures for ACS in 2020 was massive (*n* = 49, −47%), as compared with 2019 (*n* = 93) and with previous years. Using at our best all the appropriate statistical approaches evaluating possible interactions between yearly time series of the two variables, our analysis failed to demonstrate a possible relationship between these two events. Our results, therefore, refuse the initial hypothesis of a possible causative role for the reduction of air particulate matter as a determinant of the decrease in ACS during the lockdown period.

Short- and long-term exposure to air pollutants (especially PM_2.5_, but also PM_10_) has been associated with an increased risk of ACS, particularly STEMI, in previous research (4, 5). There is consistent pathophysiological background in support of such association. Indeed, fine PM incentivize inflammatory response predisposing to atherosclerosis (22), enhance thrombosis/coagulation (23), and impair endothelial function increasing plaque rupture vulnerability (24) and vessel vasoconstriction (25, 26). Mustafic et al. demonstrated a significant correlation between the rise of the most frequently evaluated components of air pollution and the occurrence of AMI (27). Subsequent studies in Europe and USA confirmed this hypothesis (4, 8, 28). Whereas, much of the recent literature supports this association, there are also studies rejecting this causal link (11–13). Different geographical context and inaccuracies in the representation of time series for both pollutants and health outcomes may have contributed in previous research to such discrepancy (5, 9, 10). We believe that the analysis of time series data including the unprecedented social upheaval represented by the lockdown measures for COVID-19 pandemic could have offered a unique chance to get further insights into this topic.

The choice to consider all the ACS in the manuscript was dictated by their common pathophysiological thread: it is actually known from previous literature data how air pollution is associated with key markers of the atherothrombotic burden, including systemic inflammatory response, oxidative stress, and endothelial dysfunction (29); this evolving process may lead to instability of vulnerable plaque, with its consequent rupture/erosion; the last one is the common hallmark of all ACS (30). Based on evidence of heart muscle damage, ACS clinically manifests as unstable angina and non-ST-elevated or ST-elevated myocardial infarction (MI) (31).

Terni is recognized as one of the most polluted cities in Central Italy (32). This is due to its particular geographical location: in fact, its basin is surrounded by the Apennine mountains; this geophysical configuration limits the dispersion and enhances the accumulation of atmospheric pollutants. The main sources of pollution are vehicular traffic, domestic heating, and industrial emissions such as a power plant for waste treatment and an active steel plant (33). More importantly, people residing in the province of Terni were found to be characterized by a very low rate of long-distance and short-distance internal migration, a situation that is completely different to the larger urban areas, such as Rome and Milan (34).

During the initial period of COVID-19 pandemic, an unprecedented massive decrease in hospital admissions for ACS (specifically −27% of STEMI and −65% of NSTEMI) was described in Italy as well as in other parts of the world (1). The real determinants of such reduction remains, to date, a matter of debate. The short-term improvement in air pollution observed during the COVID-19 crisis has been hypothesized as one of the reasons for such decrease (35). Results from our study argue against the hypothesis that the reduction in invasive procedures for ACS was at least in part explained by an improvement in air quality. Indeed, we failed to find a causative relationship between the two events even when accounting for possible associations occurring in previous years. The Granger causality test proved to be a powerful ally in this scenario, as it is aimed at identifying directed functional (“causal”) interactions from time-series data (18). Granger causality implements a statistical, predictive notion of causality whereby causes precede, and help predict, their effects; it is defined in both the time and frequency domains, and it allows for the conditioning out of common causal influences. One of its known limitations (36) is that reducing the number of observations causes uncertainty in the Granger estimates, possibly leading to rejection of the null hypothesis (no causation) due to lack of power. Therefore, we cannot exclude that the absence of any significant association between PM_2.5_ and PM_10_ air exposure and the number of invasive procedures for ACS, as observed in our single-center study, was partially driven by a low sample size (a basin of about 226,000 citizens). Moreover, given the low number of public monitoring stations for air pollutants (*n* = 6) scattered throughout the territory of the province of Terni, we acknowledge that an increased number and a more spread out distribution of such stations could ideally provide more robust data about the real distribution of air pollution determinants. However, as previously reported, the particular geographical context, the specific migration characteristics that tend to keep the population relatively stable over years, and the high levels of exposure to air pollutants could, at least in part, counterbalance such limitations.

Since data of PM_2.5_ and PM_10_ concentrations were the only air pollutants expressed in absolute terms from the list of pollutants made publicly available on the public website, we cannot exclude that other air pollutants could reflect a different behavior. Moreover, individual exposures to other sources of air pollution, such as indoor exposure and distance from busy roads, were not analyzed in this particular research project. We cannot also exclude that the air cleaning time was too short to lead to a significant causal drop in ACS during lockdown or, in parallel, that the real incidence of ACS during the COVID-19 outbreak was underestimated. Indeed, this latter finding may be caused by the fact that some patients did not make use of hospital care during the pandemic due to fear of contagion from COVID-19 (37). Due to its retrospective nature, any relationship of causation between a variable exposure and events cannot be definitely drawn from our study. Nevertheless, we adopted rather robust statistical methodology that could represent an appropriate and up-to-date analytical approach on a topic that is not yet clear, provided that further longitudinal studies are needed to better clarify this point. The lack of association between air pollution and ACS in our manuscript may be explained considering other factors; in fact, apart from environmental conditions, different triggers of acute myocardial infarction have been examined in several studies; among these, intense exercise or physical exertion, emotional stress, and inadequate control of cardiovascular risk factors can be taken into account (38). Therefore, it is plausible that some behaviors seen during the lockdown (a slight increased physical activity, especially for bodyweight training, more relaxation and family time, adherence to the Mediterranean diet and therapy, and a mild percentage reduction in smokers) may have played a role in the minor incidence of ACS (37, 39, 40).

In conclusion, we failed to find an association between an air cleaning effect observed during lockdown and a concomitant reduction in invasive procedures for ACS in the province of Terni. Reasons other than reduced air pollution should be sought to explain the observed decrease in ACS procedures.

## Data Availability Statement

The raw data supporting the conclusions of this article will be made available by the authors, without undue reservation.

## Author Contributions

AA, MD, NB, MT, GV, and GP: made substantial contributions to the conception or design of the work to the acquisition, analysis and interpretation of data for the work, drafted the work and revised it critically for important intellectual content, gave final approval of the version to be published, and agreed to be accountable for all aspects of the work in ensuring that questions related to the accuracy or integrity of any part of the work are appropriately investigated and resolved. All authors contributed to the article and approved the submitted version.

## Conflict of Interest

The authors declare that the research was conducted in the absence of any commercial or financial relationships that could be construed as a potential conflict of interest.
